# Corrosion Behavior of High-Mn Austenitic Fe–Mn–Al–Cr–C Steels in NaCl and NaOH Solutions

**DOI:** 10.3390/ma14020425

**Published:** 2021-01-16

**Authors:** Juan Bosch, Ulises Martin, Willian Aperador, José M. Bastidas, Jacob Ress, David M. Bastidas

**Affiliations:** 1 Department Chemical, Biomolecular, and Corrosion Engineering, National Center for Education and Research on Corrosion and Materials Performance, NCERCAMP-UA, The University of Akron, 302 E Buchtel Ave, Akron, OH 44325-3906, USA; jb394@zips.uakron.edu (J.B.); um11@zips.uakron.edu (U.M.); jtr45@zips.uakron.edu (J.R.); 2 Department of Engineering, Universidad Militar Nueva Granada, Carrera 11 No. 101-80, Bogota 6343200, Colombia; william.aperador@unimilitar.edu.co; 3 National Center for Metallurgical Research (CENIM), CSIC, Ave. Gregorio del Amo 8, 28040 Madrid, Spain; bastidas@cenim.csic.es

**Keywords:** high-Mn TWIP steel, pitting corrosion, chlorides, alkaline, electrochemical impedance spectroscopy, interfacial capacitance

## Abstract

The corrosion behavior of austenitic Fe–Mn–Al–Cr–C twinning-induced plasticity (TWIP) and microband-induced plasticity (MBIP) steels with different alloying elements ranging from 22.6–30 wt.% Mn, 5.2–8.5 wt.% Al, 3.1–5.1 wt.% Cr, to 0.68–1.0 wt.% C was studied in 3.5 wt.% NaCl (pH 7) and 10 wt.% NaOH (pH 14) solutions. The results obtained using potentiodynamic polarization and electrochemical impedance spectroscopy (EIS) techniques, alongside optical microscopy analysis, revealed pitting as the dominant corrosion mechanism in high-Mn TWIP steels. An X-ray diffraction analysis of the surface revealed that the main corrosion products were hematite (Fe_2_O_3_), braunite (Mn_2_O_3_), and hausmannite (Mn_3_O_4_), and binary oxide spinels were also identified, such as galaxite (MnAl_2_O_4_) and jacobsite (MnFe_2_O_4_). This is due to the higher dissolution rate of Fe and Mn, which present a more active redox potential. In addition, a protective Al_2_O_3_ passive film was also revealed, showing enhanced corrosion protection. The highest corrosion susceptibility in both electrolytes was exhibited by the MBIP steel (30 wt.% Mn). Pitting corrosion was observed in both chloride and alkaline solutions.

## 1. Introduction

A single austenite phase steel that is stable at room temperature (RT) whose deformation mechanism, defined by the stacking fault energy (SFE), consists of the gliding of individual dislocations is known as twinning-induced plasticity (TWIP) steel [1,2,3,4,5]. However, the transformation of these twins can lead to local inhomogeneity, limiting the range of applications due to their increased susceptibility to hydrogen embrittlement [6,7]. Furthermore, the high content of Mn in these alloys severely impacts the corrosion performance due to the high dissolution rates observed for Mn [8]. Additionally, the straining process induces the formation of crystallographic microbands. Microbands consisting of geometrically necessary dislocations led to a high total dislocation density state during deformation, resulting in continuous strain hardening. This microband-induced plasticity (MBIP) is the origin of the enhanced mechanical properties of the steel [9,10].

Due to the needs of lightweight materials for the automotive industry in order to reduce both CO_2_ emissions and improve fuel efficiency, special efforts has been made in alloy selection [5,6]. An austenitic TWIP steel alloy containing between 12–30 wt.% Mn was developed. The properties of this alloy perfectly match the requirements, as it is low density, has excellent mechanical properties, and has a good compromise between ductility and toughness [11,12]. This is due to the high energy absorption required during an impact event [13]. Furthermore, the alloy design allows us to obtain a stable austenitic structure in a wide range of deformation temperatures from −100 °C to +100 °C, proving the high stability of the austenite, with the stacking fault energy (SFE) being the main parameter defining the active deformation mechanism [3,14,15].

The development of low-cost Fe–Mn–Al alloys that have both good mechanical properties and high corrosion resistance has become a paramount challenge. In TWIP steels, the main alloying element is manganese, which is necessary for preserving an austenitic structure as well as maintaining a low SFE that allows mechanical twinning yet is high enough to avoid the martensitic transformation of the system [11,16,17,18,19]. However, a lower corrosion resistance has been observed for high-Mn TWIP, which might be attributed to the Mn forming an unstable oxide and thus reducing the corrosion resistance [20]. Furthermore, the content of Al and Si alloying elements affects pitting corrosion; pitting is hindered as the content of these elements increases. The presence of Al imparts better corrosion protection, developing a stable and compact passive oxide layer (Al_2_O_3_) that hinders the electrode reaction [21,22]. The presence of Si alloying element (2.8 wt.%) promotes the formation of a passive layer containing fine grains, decreasing the corrosion rate as the Si content increases (up to 3%). Si also increases the passivity breakdown potential (*E*_pit_), extending the passivity region and acting as a solid solution strengthener [23,24,25]. The presence of Mo promotes the formation of a stable passive film and decelerates pitting corrosion [26,27,28]. Furthermore, molybdates (MoO_4_^2−^) provide additional oxygen anions, interfering with Cl^−^ adsorption on the metal interface, as stated by several authors [29,30]. However, a high amount of Mo or Cr can also be detrimental to the corrosion performance if Mo/Cr carbides are formed [31,32]. The automotive industry demands high corrosion resistance as well as excellent mechanical and metallurgical properties for TWIP steels [3,33,34,35,36,37,38].

The addition of Al to TWIP steels imparts numerous benefits. Al increases the SFE significantly and, therefore, favors the formation of deformation twins over martensitic transformation [9,39,40]. Furthermore, Al alloying strengthens the austenite by solid solution hardening. Aluminum additions also have proven effective in suppressing delayed fracturing in press-formed parts [40]. However, the addition of Al leads to a lower strain hardening rate, resulting in a decrease in tensile strength [40]. In contrast to Al, the presence of Si decreases the amount of austenite phase and sustains the transformation of γ-austenite to ε-martensite during cooling and deformation processes [41].

The addition of Cr to ferrous alloys promotes the formation of a passive layer, thus improving the corrosion resistance [42]. Chromium is a ferrite stabilizing element and also increases the solubility of N in the melt during casting [43]. However, the addition of Cr to the Fe–Mn-based alloy system raises the SFE [44]. Carbon can be used as an alloying element to stabilize austenite and strengthen the matrix due to solid solution hardening [45]. Manganese and Ni are gammagenous elements that favor the austenite phase. However, Mn enhances corrosion kinetics due to its high anodic dissolution rate [8]. Binary Ni–P alloy coatings can substantially improve the corrosion performance and surface hardness of Fe–25Mn–3Al TWIP steel alloys compared to uncoated TWIP steel [46].

The motivation of this study arose from the need for the further understanding of microalloying processes in order to improve the corrosion behavior of high-Mn steels. This work aims to reveal the corrosion mechanism of high-Mn austenitic Fe–Mn–Al–Cr–C steels with compositions ranging from 22.6% to 30 wt.% Mn, 5.2–8.5 wt.% Al, 3.1–5.1 wt.% Cr, and 0.68–1.0 wt.% C. Additionally, one specimen containing an extra 2.8 wt.% Si and another containing 1.1 wt.% Mo were studied in 3.5 wt.% NaCl and in 10 wt.% NaOH solutions using electrochemical corrosion monitoring. Potentiodynamic polarization (PP) and electrochemical impedance spectroscopy (EIS) techniques were utilized. A corrosion product analysis was carried out with the X-ray diffraction (XRD) and optical microscopy (OM) techniques.

## 2. Materials and Methods

High-Mn TWIP steel specimens were obtained by the melting process of high-purity metals in an induction furnace under a protective argon gas environment. Ingots were forged at 1100 °C for 2 h and were homogenized at 1100 °C for 11 h. The ingots were then subjected to a thermal aging treatment at 600 °C for 18 h, followed by cooling in air at RT. The ingots were cut into slices that were 2.5 ± 0.2 mm thick using electro-discharge machining. Finally, the resulting slices were annealed at 1100 °C for 1 h in a tubular furnace with a constant flux of argon and cooled in air at RT. To determine the elemental composition, an S8 Tiger wavelength dispersive spectrometer was used. Table 1 shows the results from the X-ray fluorescence (XRF) for the three high-Mn austenitic steels studied and the SFE values obtained using stacking fault energy maps, as reported elsewhere [10].

In order to reveal the corrosion products of the TWIP steels after exposure, the samples were first epoxy mounted and then polished on a Struers Polisher Tegramin-30 following standard metallographic procedures. Specimens were ground up to 1200 grit using silicon carbide paper to remove native oxides and polished to a mirror finish using a colloidal silica suspension solution of 0.05 µm. Finally, samples were degreased in ethanol and air-dried.

Two different electrolyte test solutions were used—a neutral (pH 7) 3.5 wt.% NaCl solution and an alkaline (pH 14) 10 wt.% NaOH solution—to study the different corrosion behavior depending on the pH of the electrolyte [47]. Electrochemical measurements were performed using a flat electrochemical cell consisting of 3 electrodes: a working electrode which was the test specimen, the reference electrode which was a saturated calomel electrode (SCE), and a platinum auxiliary electrode. Triplicate measurements were obtained for all tests in a Gamry Reference 600 potentiostat. The corrosion potential (*E*_corr_) value was obtained after steady state was reached at 25 °C. Afterwards, PP tests were recorded in a potential range between −200 mV < *E*_corr_ < +1200 mV versus SCE at a scan rate of 0.1667 mV s^−1^ according to the ASTM G61-86 standard [48]. In addition, EIS tests were recorded after a 60 min relaxation time to stabilize the *E*_corr_, with an AC excitation signal of 10 mV_rms_. A frequency scan range between 10^5^ and 10^−2^ Hz was analyzed, acquiring 7 points per decade, following ASTM G106‒89 standard [49].

Microstructural and corrosion product characterization was carried out by optical microscopy (OM) with an Olympus BX 51 TF. A crystallographic analysis was performed using X-ray diffraction (XRD) in a diffractometer equipped with a goniometer PW3050/60 (θ/θ) and an XPERT-PRO system. XRD patterns were obtained using a monochromatic radiation of Cu Kα with wavelength 1.54 Å, working at 40 kV and 40 mA, in a 2θ scan range from 20.01° to 99.99°, with a step size of 0.02°/2θ, and at one step per 1 s. The XRD analysis of the TWIP and MBIP specimens was performed after the electrochemical corrosion tests to elucidate the composition of the oxide layer without removing the corrosion products. XRD peaks were assigned using the Powder Diffraction File (PDF) cards from the International Center for Diffraction Data (ICDD).

## 3. Results and Discussion

### 3.1. XRD Analysis

**Figure 1:**
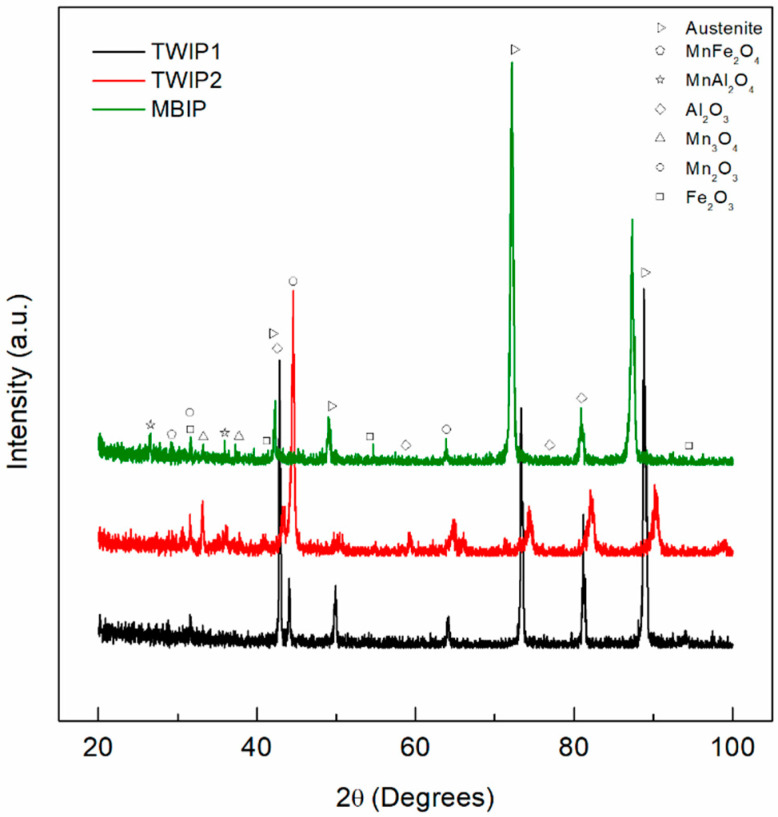
XRD pattern of the high-Mn steels after exposure to 3.5 wt.% NaCl at RT for TWIP1 (Fe–22.6Mn–6.3Al–3.1Cr–0.68C), TWIP2 (Fe–28Mn–5.2Al–5.1Cr–2.8Si–0.95C), and microband-induced plasticity (MBIP) (Fe–30Mn–8.5Al–3.2Cr–1.1Mo–1.0C).

**Figure 2:**
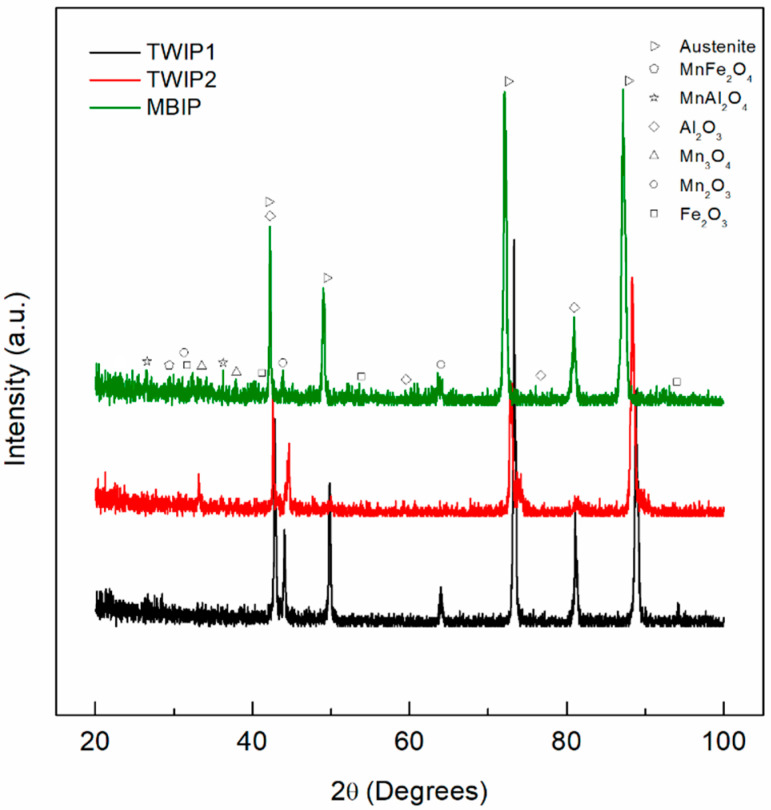
XRD pattern of the high-Mn steels after exposure to 10 wt.% NaOH at RT for TWIP1 (Fe–22.6Mn–6.3Al–3.1Cr–0.68C), TWIP2 (Fe–28Mn–5.2Al–5.1Cr–2.8Si–0.95C), and MBIP (Fe–30Mn–8.5Al–3.2Cr–1.1Mo–1.0C).

XRD patterns depict the presence of different corrosion products compounds formed on the three high-Mn steels. In Figure 1, the diffraction patterns obtained after exposure to 3.5 wt.% NaCl solution are shown, while Figure 2 corresponds to 10 wt.% NaOH solution. The main corrosion products found were hematite (Fe_2_O_3_, PDF 33-0644), bixbyite (Mn_2_O_3_, PDF 24-0735), and hausmannite (Mn_3_O_4_, PDF 24-0734). In addition, binary oxide spinels were identified, including galaxite (MnAl_2_O_4_, PDF 29-0880) and jacobsite (MnFe_2_O_4_, PDF 75-0034). The spontaneous formation of an Al_2_O_3_ protective film was also revealed (α-Al_2_O_3_, PDF 83-2080). The austenitic structure of the high-Mn TWIP was also evidenced by the presence of diffraction peaks at 2θ angles of 42°, 50°, 73°, and 89° (γ-Fe, PDF 65-4150).

### 3.2. Microstructure and Surface Characterization

**Figure 3:**
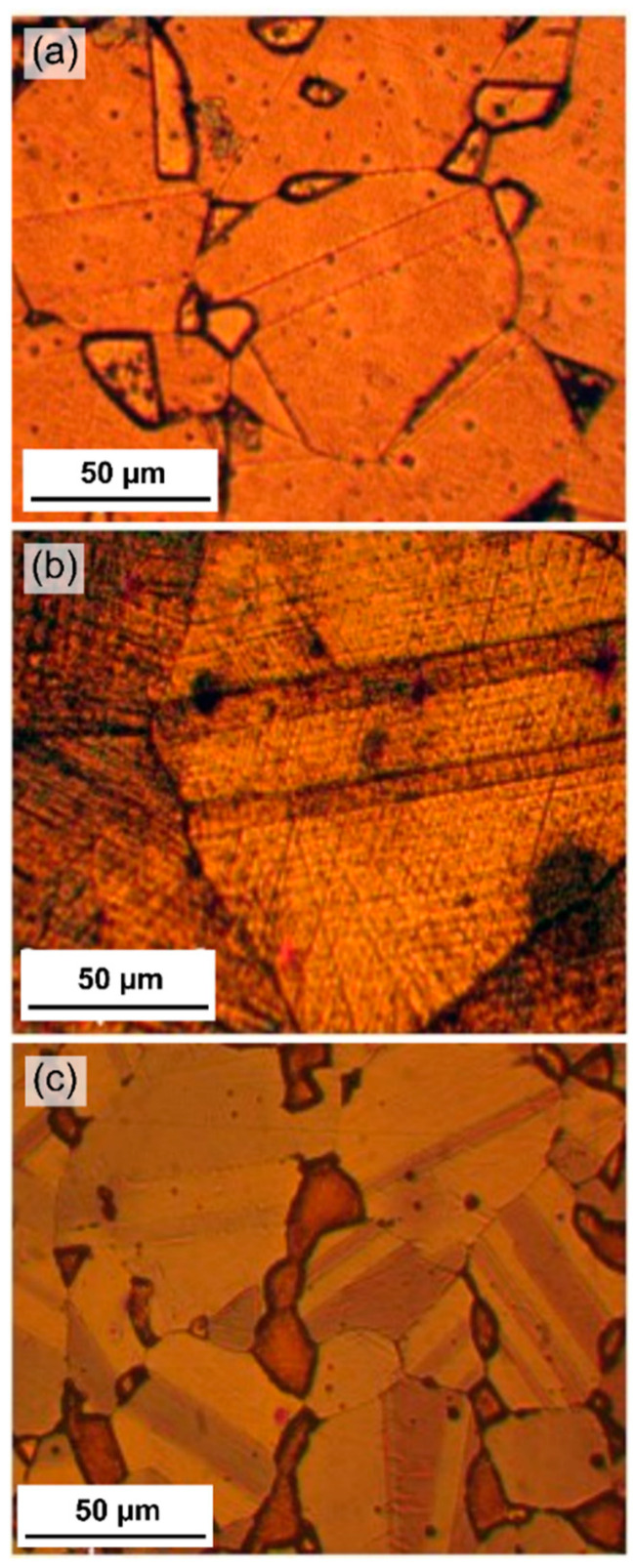
Optical microstructure analysis of the three high-Mn steels studied: (**a**) TWIP1, (**b**) TWIP2, and (**c**) MBIP.

**Figure 4:**
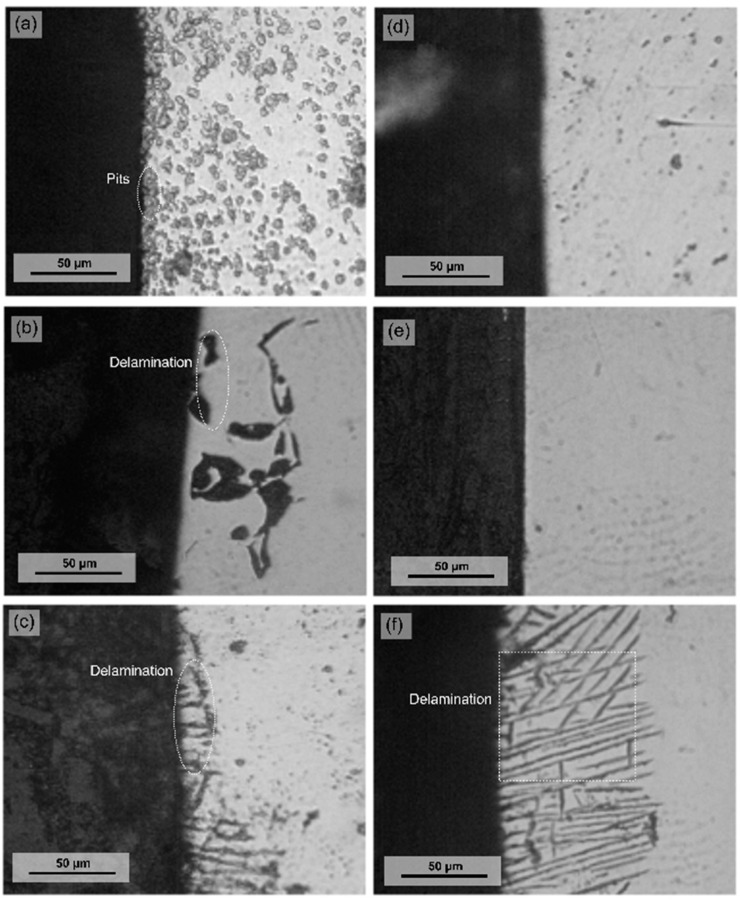
Optical cross-section micrograph for (**a**) TWIP1, (**b**) TWIP2, and (**c**) MBIP steels in 3.5 wt.% NaCl solution, and (**d**) TWIP1, (**e**) TWIP2, and (**f**) MBIP steels in 10 wt.% NaOH solution.

The microstructure characterization by OM is presented in Figure 3a–c, which depict the austenitic structure of the three high-Mn steels. The OM cross-section micrographs for the three alloys TWIP1, TWIP2, and MBIP, exposed to the 3.5 wt.% NaCl and 10 wt.% NaOH electrolytes, are presented in Figure 4. For the samples exposed to the chloride environment shown in Figure 4, the most active alloy was the TWIP1 due to the severe pitting corrosion exhibited. This can be explained by the lower contents of Mo and Si that this alloy contains; Si imparts passivity by the formation of intermetallic materials, such as Fe_3_Al-Si, and increases the *E*_pit_, as well as extending the passive region [21,22]. In addition, molybdenum alloying modifies the polarity of passive film by generating molybdates and also creates a bipolar interface that promotes repassivation by the deactivation of pit growth. Molybdenum inhibits corrosion through forming MoO_3_ oxide layers which convert to MoO_4_^2−^ at the electrolyte/electrode interface, preventing the flux of OH^−^ and Cl^−^ ions into surface passive films and pits [25]. In contrast, the exposure to the highly alkaline electrolyte (10 wt.% NaOH) showed a more noble *E*_corr_ compared to the samples exposed to chloride-containing solution (3.5 wt.% NaCl), in which TWIP2 is the most active alloy.

### 3.3. Electrochemical Corrosion Analysis

**Figure 5:**
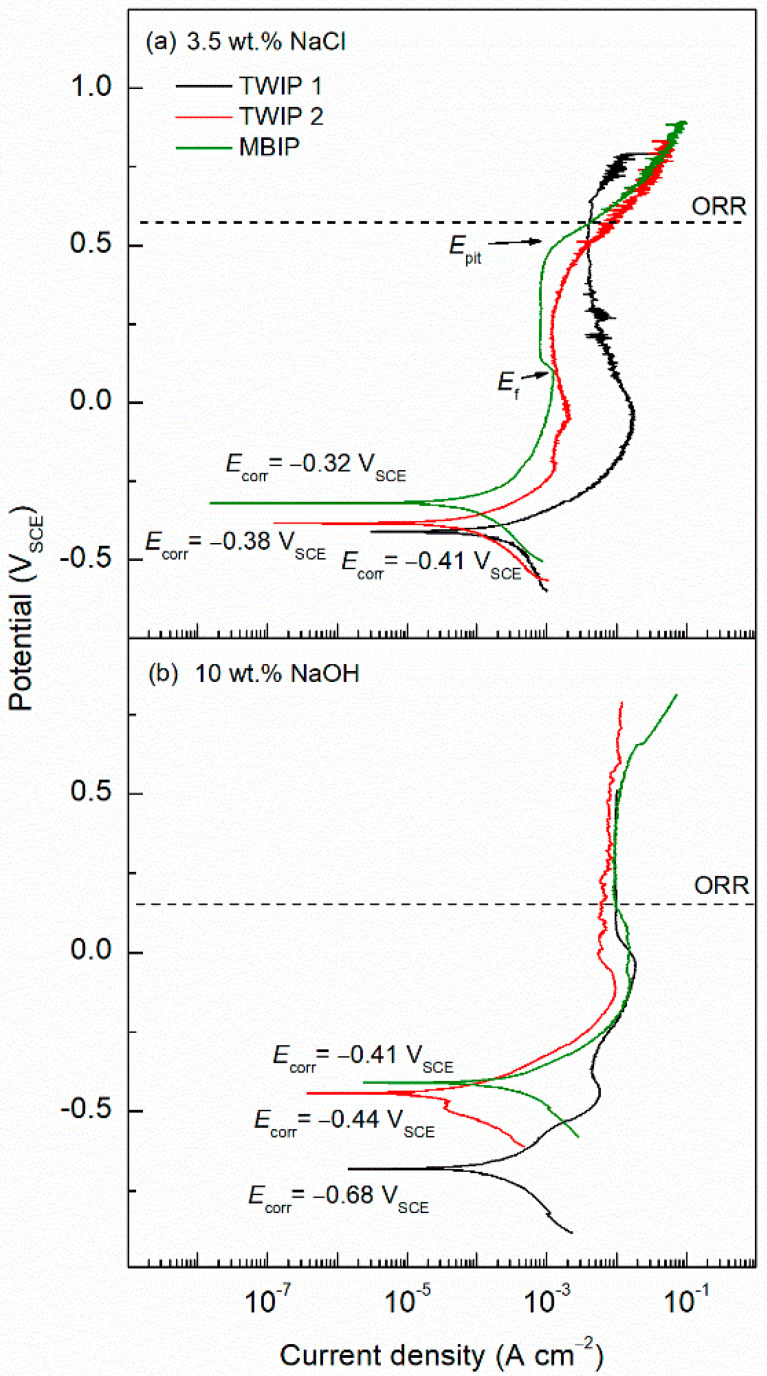
Potentiodynamic polarization curves for TWIP1, TWIP2, and MBIP in (**a**) 3.5 wt.% NaCl solution, and (**b**) 10 wt.% NaOH solution.

PP scans were performed for the three alloys TWIP1, TWIP2, and MBIP in both electrolytes, 3.5 wt.% NaCl, and 10 wt.% NaOH. The cathodic kinetics were modified by the presence of alloying elements; it was observed that the oxygen reduction reaction (ORR) in 10 wt.% NaOH was promoted with the addition of Mo, as the exchange current density values were 4.32 × 10^−8^, 5.65 × 10^−10^, and 6.06 × 10^−7^ A cm^−2^ for MBIP, TWIP1, and TWIP2, respectively. For the 3.5 wt.% NaCl solution, the exchange current density values were 3.82 × 10^−7^, 9.35 × 10^−10^, and 1.52 × 10^−8^ A cm^−2^ for TWIP1, MBIP, and TWIP2, respectively. This exchange current density was obtained with the intersection of the cathodic Tafel slope and the ORR potential, leading to a current density value associated with the exchange current density. The anodic polarization curves depict an active-to-passive transition with an ill-defined Flade potential (*E*_f_) peak (a shoulder-like shape), followed by a passive region. In the transpassive region, a breakdown pitting potential (*E*_pit_) corresponding to a sharp increase in the current density was obtained. The ORR was observed in neutral pH for potentials above 0.574 V vs. SCE according to the Nernst equation at RT (see Equation (1)):

(1) $$E_{O_{2}/{\mathrm{OH}}^{-}}=E_{O_{2}/{\mathrm{OH}}^{-}}^{ᴼ}-0.059\log\frac{\left[{\mathrm{OH}}^{-}\right]}{p_{O_{2}}^{1/4}},$$

where $E_{O_{2}/{\mathrm{OH}}^{-}}$ is the reversible potential of the oxygen; $E_{O_{2}/{\mathrm{OH}}^{-}}^{ᴼ}$ is the standard reduction potential, being 0.401 V_SHE_ for alkaline media; $\log\;\left[{\mathrm{OH}}^{-}\right]$ is a function of the pH; $p_{O_{2}}^{1/4}$ is the partial pressure of O_2_ and equals 1 atm. This ORR is observed once the potential increases above the oxygen equilibrium line at 0.574 V vs. SCE for the neutral pH and 0.160 V_SCE_ for the alkaline media. For increased potentials, the ORR rate also increases as more oxygen is reduced. This causes the nucleation of metastable pitting in the surface of the metal. The higher the anodic potentials above the oxygen equilibrium line, the higher the density of metastable pits, this can be seen in Figure 5a as wider and more frequent current shifts (metastable pitting, dissolution followed by repassivation). Surpassing the oxygen equilibrium line implies an enhanced dissolution rate, higher than the repassivation and the passive film growth, thus promoting the nucleation of stable pits. As more metastable pits grow, the chances for these pits to connect and form stable pits are increased, promoting the dissolution of the metal. The order of the anodic current passivity (*i*_pass_) plateau was MBIP < TWIP2 < TWIP1, thus the passive layer of MBIP is the most stable, which may be attributed to the high passivating properties of the Mo alloying element [50] and of Al, which are higher that those of Cr and considerably higher than those of Mn and Fe [21,51,52]. Oscillations in the current density can be observed for the TWIP1 alloy in the beginning of the passive region and after the *E*_f_ associated with metastable pitting (see Figure 5a). Hamada and Karjalainen reported that high-Al (8 wt.%) TWIP steel exhibited current density oscillations in the beginning of the passive region attributed to salt-film precipitation as a passivation mechanism, contrary to the spontaneous oxide film formation in a Cr-bearing steel [21]. The TWIP2 alloy improves the passivating properties with respect to the reference steel (TWIP1). A low Cr content enhances the passivity of the material. However, a high content (6–9 wt.%) has a detrimental effect on corrosion resistance [31]. This is because Cr causes phase-segregations, generating nucleation points for pitting corrosion [37]. Finally, the TWIP2 alloy also shows small current oscillations or the generation of metastable pits in the passive region (see Figure 5a). Using the Tafel extrapolation method, the corrosion current density (*i*_corr_) and the Tafel slopes were determined, 2.63 × 10^−4^, 5.41 × 10^−5^, and 4.34 × 10^−4^ A cm^−2^ for TWIP1, TWIP2, and MBIP, respectively, in 3.5 wt.% NaCl solution, as can be seen in Table 2. Using Faraday’s law in Equation (2) (using 11.6 as the conversion factor), the corrosion rate was determined, yielding 0.00243, 0.00063, and 0.005 mm year^−1^ values for TWIP1, TWIP2, and MBIP, respectively:

(2) $$\mathrm{CR}=\frac{i_{\mathrm{corr}}\;K\;\mathrm{EW}}{\rho \;A},$$

where CR is the corrosion rate, $i_{\mathrm{corr}}$ is the corrosion current density, K is a constant that defines the units of CR (equal to 3272 to obtain mm/year), EW is the equivalent weight in grams/equivalent, ρ is density, and A is the exposed area in cm^2^.

The increase in Al content yielded an increase in *E*_corr_ and a decrease in *i*_pass_, whereas the increase in Mn alloying element did not produce a negative effect on the *i*_pass_, as can be seen in Figure 5b. This anomalous behavior, without a negative influence of an Mn content increase on the corrosion resistance, may be attributed to the amphoteric properties of the Al alloying element, which generates an unstable oxide layer in the alkaline (pH 14) 10 wt.% NaOH solution [36]. According to the Pourbaix diagram, Al generates a protective and stable Al_2_O_3_ film in the 4–9 pH range [53]. As indicated above, the passive layer of Fe–Mn–Al–Cr steel is stable due to the spontaneous passivation the Al_2_O_3_ film imparts, which is higher than Cr, Mn, and Fe [21,51]. In the case of the MBIP steel, the corrosion resistance may be enhanced by the 1.1 wt.% Mo alloying element (see Table 1), stabilizing the passive layer [50].

Using the Tafel extrapolation method, the corrosion current density (*i*_corr_) and the Tafel slopes were determined. The *i*_corr_ was 1.48 × 10^−4^, 2.65 × 10^−5^, and 1.27 × 10^−4^ A cm^−2^ for TWIP1, TWIP2, and MBIP, respectively, in 10 wt.% NaOH. The Tafel slopes presented in Table 2 showed higher cathodic kinetics, indicating the overall electrochemical reaction was controlled by the oxygen reduction reaction at the cathode, and the steady-state limiting current density was achieved around 10^−2^ A cm^−2^, corresponding to the maximum diffusion current density in the oxygen reduction reaction (ORR). The corrosion rates were 0.00172, 0.00031, and 0.0015 mm year^−1^ for the TWIP1, TWIP2, and MBIP, respectively; this is in accordance with the results observed in the OM, showing lower corrosion rates for the samples exposed to 10 wt.% NaOH. The electrochemical transfer coefficients can be obtained and discussed using Equations (3) and (4) [54]:

(3) $$\alpha_{a}=\frac{\mathrm{RT}}{nF}\frac{1}{\beta_{a}},$$

(4) $$\alpha_{c}=-\frac{\mathrm{RT}}{nF}\frac{1}{\beta_{c}},$$

where α_a_ and α_c_ are the anodic and cathodic transfer coefficients, respectively; R is the ideal gas constant (8.314 J mol^−1^ K^−1^); T is the temperature in K; *n* is the number of electrons; F is the Faraday constant (96,485 C mol^−1^); and *β*_a_ and *β*_c_ are the anodic and cathodic Tafel slopes in V/dec, respectively. Table 2 summarizes the electrochemical parameters obtained by PP, showing the highest charge transfer coefficient for MBIP in 10 wt.% NaOH, while TWIP1 presents the highest in 3.5 wt.% NaCl. In the case of the TWIP2 steel, the α_a_ and α_c_ values are in the same range independently of the electrolyte, thus indicating a passive-like behavior.

The low value of 0.00031 mm year^−1^ for the TWIP2 steel may be attributed to the low presence of Al (5.2 wt.%) and the high Cr content (5.1 wt.%). As indicated previously, this higher Cr content enhances the corrosion resistance [37]. Moreover, the presence of Si (2.8 wt.%) has a beneficial influence on the corrosion resistance, which favors the formation of a stable passive film and increases the *E*_pit_. Finally, the passivation mechanism of the three high-Mn steels tested in the 10 wt.% NaOH solution may be due to the spontaneous oxide layer formation in Al-bearing steels.

**Figure 6:**
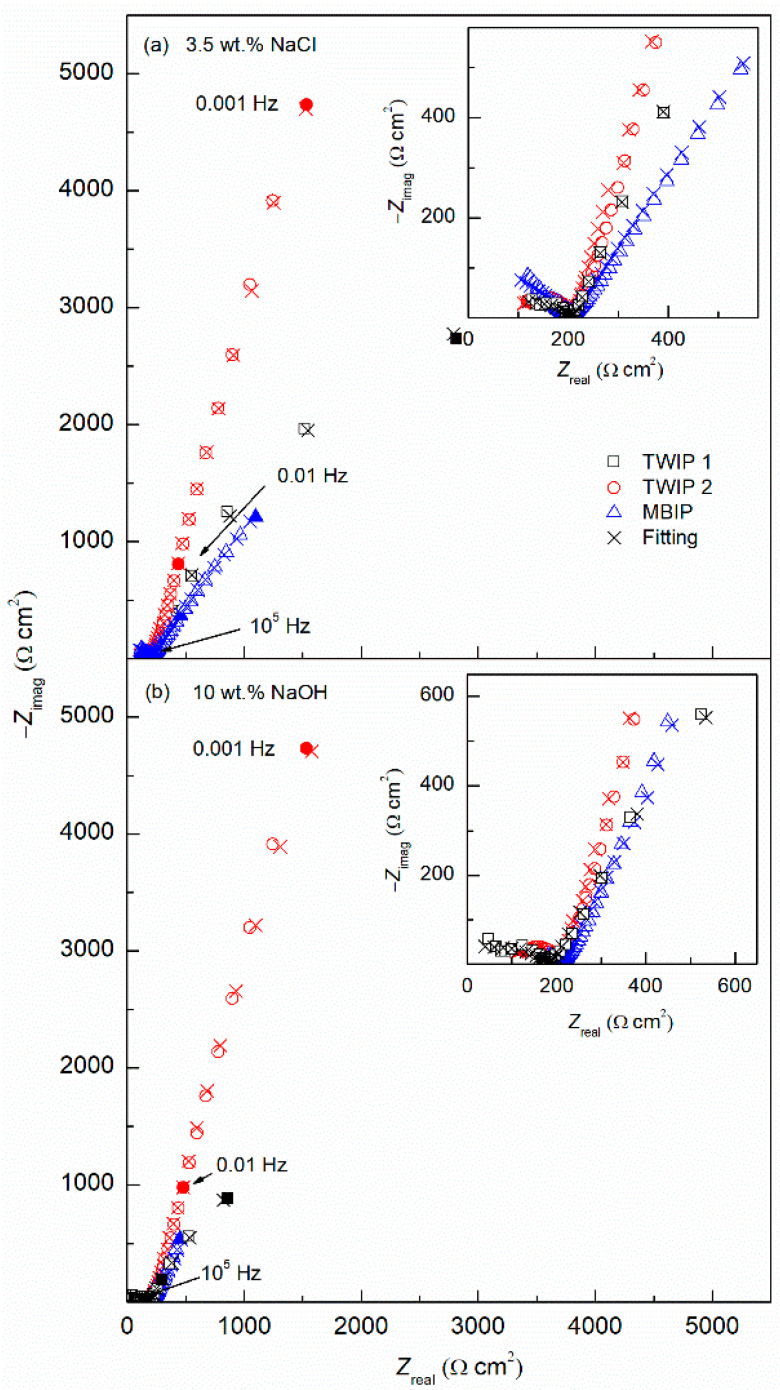
Nyquist plots obtained for TWIP1, TWIP2, and MBIP steels in (**a**) 3.5 wt.% NaCl, and (**b**) 10 wt.% NaOH solutions.

**Figure 7:**
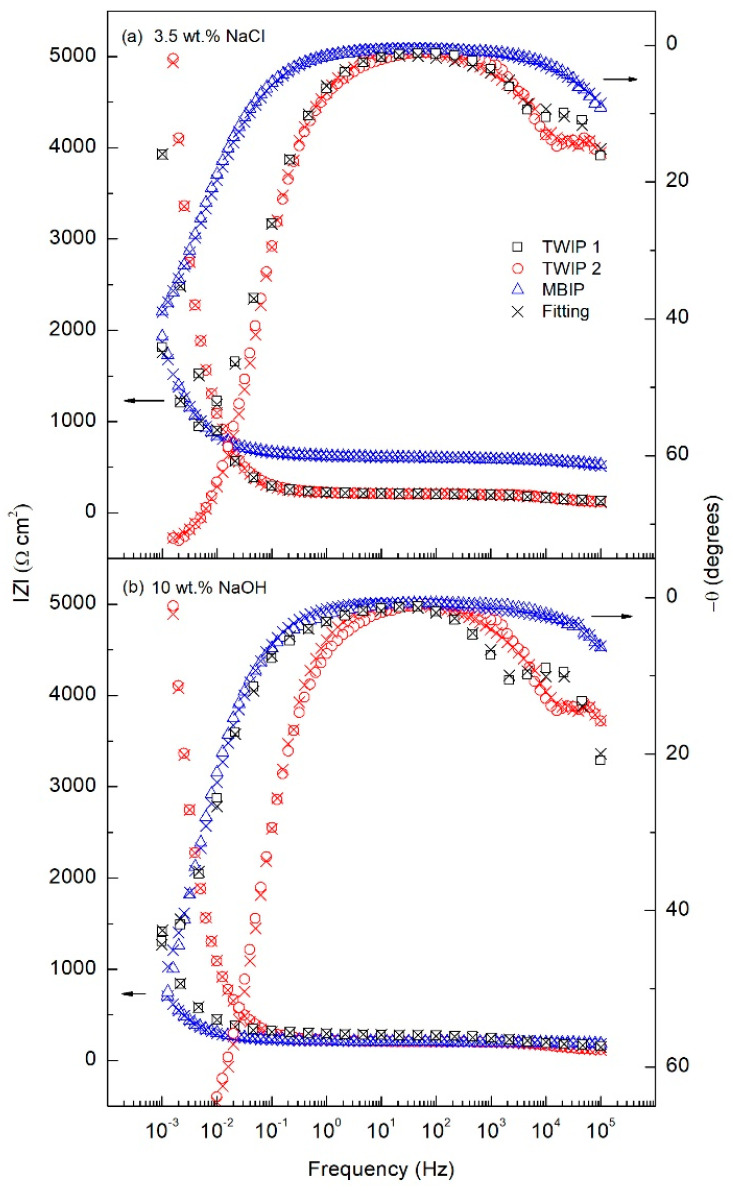
Bode plots obtained for TWIP1, TWIP2, and MBIP steels in (**a**) 3.5 wt.% NaCl, and (**b**) 10 wt.% NaOH solutions.

**Figure 8:**
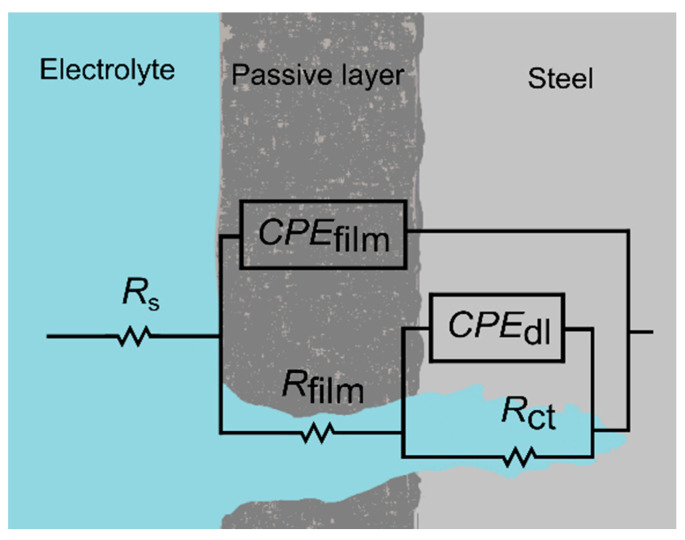
Electrical equivalent circuit (EEC) used to fit the impedance data.

Figure 6 represents the Nyquist plots for the three tested high-Mn steel alloys, where Figure 6a shows the corrosion behavior in 3.5 wt.% NaCl solution and Figure 6b in 10 wt.% NaOH solution. Two semicircles are observed, which correspond to the passive film and double-layer interfaces. The Bode plots are presented in Figure 7, a two-time constants electrochemical process is shown, identified by the phase angle inflexion. The EIS results were modeled by the electrical equivalent circuit (EEC) of Figure 8 to describe the electrochemical properties of the interfaces of high-Mn TWIP steels. The EIS results obtained in both corrosion media, chloride and alkaline, were modeled with the same EEC. The EEC depicted in Figure 8 is composed of two time-constants (*R*//*CPE*) representing a hierarchically distributed circuit, where the first time-constant, found at high frequencies, corresponds to the passive film-electrolyte interface (*R*_film_//*CPE*_film_), where a non-uniform current distribution along the interface is denoted by the constant phase element of the passive film (*CPE*_film_) and the resistance of the passive film (*R*_film_), and the second time constant, found at low frequencies corresponds to the electrochemical double layer represented by (*CPE*_dl_), including the charge transfer resistance (*R*_ct_), which is inversely proportional to the corrosion rate (*v*_corr_), and denotes an electrode process (*R*_ct_//*CPE*_dl_) [55,56,57]. The ECC also accounts for the solution ohmic drop denoted by the electrolyte resistance (*R*_s_). The *CPE* accounts for the non-ideal capacitance response of an active electrode, and its impedance is described by *Z*_CPE_ = (*Y*)^−1^(*jω*)^−*n*^, where *Y* is the admittance (S cm^−2^ s^n^), *ω* is the angular frequency (rad s^−1^), *j* is the imaginary number, and *n* is a dimensionless variable that represents the non-ideality of the *CPE*. The values of *n* range from 1, which would make the *CPE* an ideal capacitor, to 0 which will represent an ideal resistor and, where values of 0.5 could show a diffusion process represented by a Warburg element [38,56,57,58,59,60].

**Figure 9:**
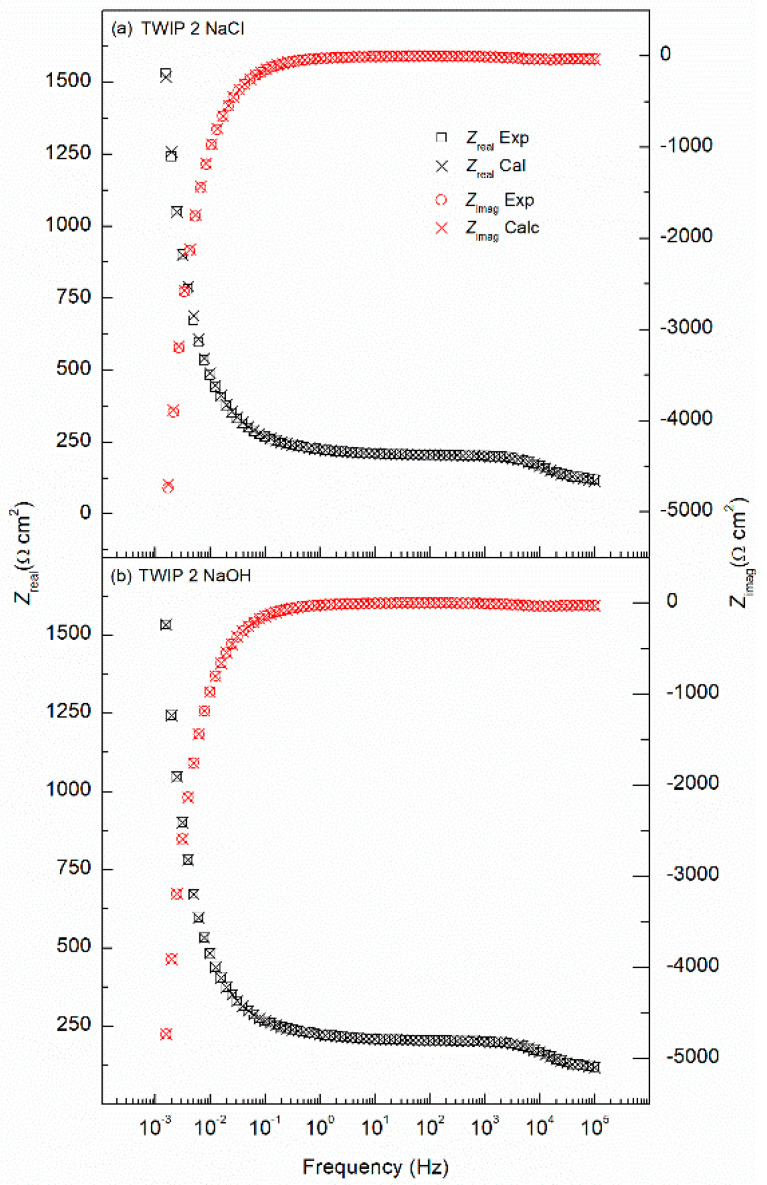
Comparison of the experimental impedance data for TWIP2 in (**a**) 3.5 wt.% NaCl solution, (**b**) 10 wt.% NaOH solution, and the impedance data calculated using the Kramers–Kronig (KK) transformations.

The Nyquist plots show a good fitting, as the experimental data, denoted by empty symbols, and the calculated data using EEC and denoted by crosses present a consistent matching for all three alloys in both solutions. Table 3 summarizes all the values from the EEC elements, where the error of each parameter was kept under 10% and the goodness of fit (*χ*^2^) was in 10^−3^ for all the fitting curves. In addition, the robustness of the experimental impedance data was evaluated using the Kramers–Kronig (KK) transforms [58,61]. Figure 9 shows an example of the comparison between the experimental EIS data from Figure 6 for the TWIP1 steel in the 3.5 wt.% NaCl solution and in the 10 wt.% NaOH solution and those obtained by means of the KK transforms. The correlation between the experimental and the KK-transformed data is excellent as can be seen in Figure 9, confirming the robustness of the EIS data and, therefore, its validity to model the high-Mn TWIP steel electrochemical corrosion process.

*R*_s_ values of 26.0–28.1 and 30.1–32.1 Ω cm^2^ are obtained for all three high-Mn steels in both electrolytes, 3.5 wt.% NaCl, and 10 wt.% NaOH solution, respectively. Thus, this shows that the electrolyte conductivity is similar. The *R*_film_ stays in the same order of magnitude for both solutions, slightly changing between alloys ranging from 142.5 to 607.7 Ω cm^2^ for the NaCl solution and from 136.6 to 295.1 Ω cm^2^ for the NaOH solution. The pseudo-capacitance of the surface oxide film (*Y*_film_) is in the µS cm^−2^ s*^n^*^1^, which is in the typical order of magnitude for passive films, for both solutions, being higher in the NaOH than in the NaCl by almost double the value, indicating a thinner film in the case of the NaOH. Accordingly, *n*_1_ remains withing the same range from 0.492 to 0.588 for the NaCl and from 0.466 to 0.607 for the NaOH. The *R*_ct_ in NaCl solution significantly changes between samples, having the highest impedance value, thus more protective, for TWIP2 with 21593 Ω cm^2^, followed by TWIP1 and MBIP with 2220 and 952 Ω cm^2^, respectively. There is a reduction of one order of magnitude among TWIP2 and TWIP1, and a further order of magnitude reduction among TWIP1 and MBIP. This same trade is seen in the NaOH solution where TWIP2, TWIP1 and MBIP have 11310, 3324 and 555 Ω cm^2^ respectively. Finally, the *Y*_dl_ and *n*_2_, remain barely unchanged from sample to sample and solution, being in the 0.01 µS cm^−2^ s*^n^*^2^ order of magnitude and 0.711 to 0.847 range, respectively.

The *i*_corr_ was estimated through the Stern–Geary relationship, *i*_corr_ = *B*/*R*_ct_, where *B* is a constant defined by *B* = (*β*_a_*β*_c_)/2.3(*β*_a_ + *β*_c_), *β*_a,_ and *β*_c_ are the anodic and cathodic Tafel slopes, respectively, in the PP tests (Figure 5a,b), *B* values can be seen in Table 2. Finally, *R*_ct_ has been defined above; see Table 2. The corrosion rate determined using Faraday’s law was 0.27, 0.01, and 0.67 mm year^−1^ for TWIP1, TWIP2, and MBIP in 3.5 wt.% NaCl solution, respectively, and 0.23, 0.03, and 0.29 mm year^−1^ for TWIP1, TWIP2, and MBIP in 10 wt.% NaOH solution, respectively. These findings are higher than the corrosion rate results yielded by the PP experiments of Figure 5. Furthermore, the worst performing alloy was found to be MBIP, which is in accordance with PP curves. This might be due to the effect of Mn, promoting a higher dissolution rate.

As previously described, *CPE* is a non-ideal element which represents a branched ladder R//C network, and hence it is not possible to directly measure the capacitance. In order to get the effective capacitance (*C*_eff_), the resistance has to be also taken into account for correcting the value. The equation proposed by Brug et al. enables the calculation of the accurate value of the *C*_eff_ of the double layer (*C*_eff,dl_), which uses the resistance of the *R*_ct_ and the *R*_s_ (see Equation (5)) [59].

(5) $$C_{\mathrm{eff,dl}}={\left[Y_{\mathrm{dl}}{\left(\frac{1}{R_{s}}\;+\;\frac{1}{R_{\mathrm{ct}}}\right)}^{n}\right]}^{\left(\frac{1}{n-1}\right)}.$$

**Figure 10:**
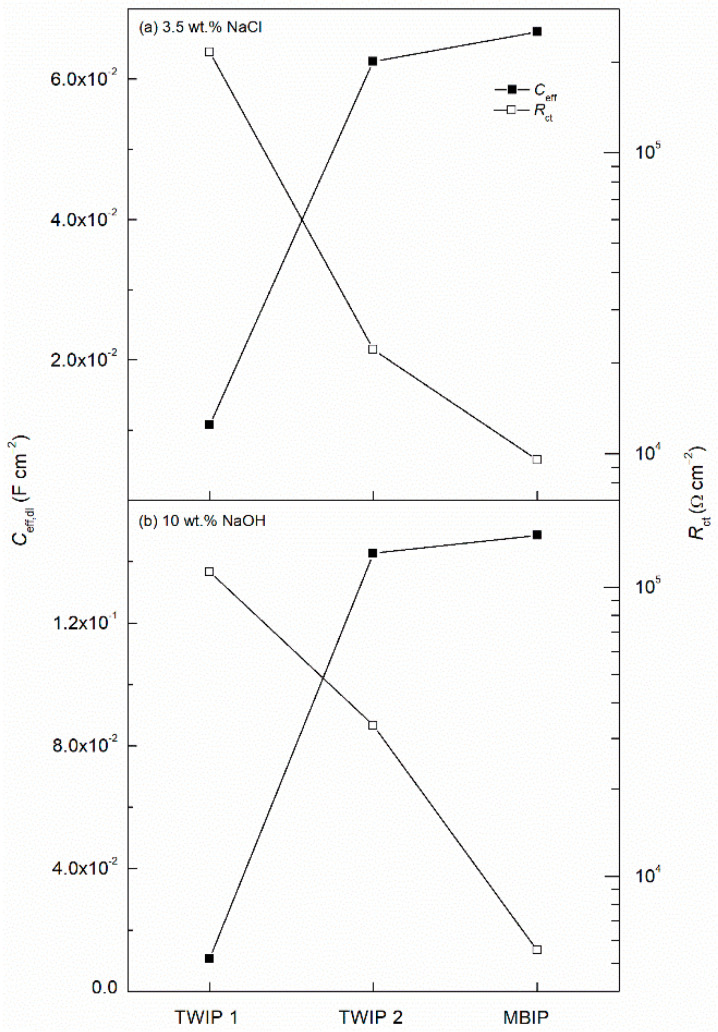
Evolution of the *C*_eff,dl_ and *R*_ct_ parameters for TWIP1, TWIP2, and MBIP steels in (**a**) 3.5 wt.% NaCl, and (**b**) 10 wt.% NaOH solutions.

The obtained values for the *C*_eff,dl_ and *R*_ct_ parameters for the three high-Mn steels studied in the 3.5 wt.% NaCl and 10 wt.% NaOH solutions are depicted in Figure 10. It is observed that the higher the *R*_ct_ the lower the *C*_eff,dl_, which is in good agreement, as the higher the impedance and the lower the capacitance the more protection the film imparts [57]. The TWIP2 steel shows the lowest corrosion kinetics as it shows the highest *R*_ct_ and the lowest *C*_eff,dl_ followed by TWIP1 and MBIP, this behavior is seen in both solutions 3.5 wt.% NaCl and 10 wt.% NaOH. From the trend of decreasing the impedance and increasing the capacitance with addition of Mn in the TWIP alloy, 22.6, 28 and 30 wt.% respectively, for chloride and alkaline solutions, it can be concluded that increasing the Mn content leads to lower corrosion properties. The results from the AC and DC techniques are in good agreement and prove that the TWIP2 alloys exhibit more noble corrosion behavior.

## 4. Conclusions

- The three high-Mn steels tested showed austenitic microstructures. After electrochemical experiments, the optical micrographs showed pitting and uniform corrosion for all samples in both chloride and alkaline solutions.
- The X-ray diffraction results indicated that the main surface corrosion products identified were Fe oxide (Fe_2_O_3_ or Fe_3_O_4_), Mn oxide (MnO_2_, Mn_2_O_3_, or Mn_3_O_4_), MnAl_2_O_4_, and MnFe_2_O_4_. This is due to the higher dissolution rate of Fe and Mn, which present a more active redox potential. In addition, a passivating Al_2_O_3_ oxide film was revealed.
- The PP scans showed that the cathodic kinetics decreased as the content of Mn increased. This behavior was interpreted in terms of the catalytic properties of the alloys towards the oxygen reduction reaction (ORR). The anodic branches depicted an active-to-passive transition defining a shoulder-like followed by a passivation region, an *E*_pit_, and the evolution of oxygen. In the 10 wt.% NaOH solution, the anodic branch was stable without current density oscillations, which was attributed to the spontaneous passivation imparted by the Al_2_O_3_ film. Contrarily, in the 3.5 wt.% NaCl solution current density oscillations were observed mainly in TWIP1 and TWIP2 and were attributed to a salt-film precipitation passivation mechanism. The corrosion rate was found to be higher in both electrolytes for MBIP because of the higher Mn content, while the lowest corrosion rate was recorded for the TWIP2 due to the presence of Mo and the higher Cr content imparting passivity.
- The EIS results revealed a capacitive behavior with two-time constants, one at high frequencies for the passive layer and one at low frequencies for the corrosion process. The results are in agreement with the PP scans showing the lowest corrosion rate in TWIP2 and the highest in MBIP.

## Figures and Tables

**Table 1:** Elemental compositions of the high-Mn steels tested (wt.%), Fe balance, and stacking fault energy (SFE) (mJ m^−2^) [10].

Sample	Mn	Al	Cr	Si	Mo	C	SFE (mJ m^−2^)
TWIP1	22.60	6.30	3.10	−	−	0.68	62.2
TWIP2	28.00	5.20	5.10	2.80	−	0.95	55.1
MBIP	30.00	8.50	3.20	−	1.10	1.00	76.2

**Table 2:** Electrochemical parameters obtained from the potentiodynamic polarization curves in Figure 5, for TWIP1, TWIP2, and MBIP steels in 3.5 wt.% NaCl and 10 wt.% NaOH solutions.

Specimen	*E*_corr_ (V_SCE_)	*i*_corr_ (A cm^−2^)	*i*_pass_ (A cm^−2^)	*β*_a_ (mV/dec)	*β*_c_ (mV/dec)	*B* (mV)	*R*_p_ (Ω cm^2^)	*i*_0,ORR_ (A cm^−2^)	*α*_a_	*α*_c_
	3.5 wt.% NaCl									
TWIP1	−0.41	2.63 × 10^−4^	4.05 × 10^−3^	80.68	136.44	22.04	179.36	4.32 × 10^−8^	0.321	−0.189
TWIP2	−0.38	5.41 × 10^−5^	1.22 × 10^−3^	204.92	277.40	51.24	947.51	9.35 × 10^−10^	0.126	−0.093
MBIP	−0.32	4.34 × 10^−4^	8.46 × 10^−4^	190.37	363.30	54.31	125.03	1.52 × 10^−8^	0.135	−0.071
	10 wt.% NaOH									
TWIP1	−0.68	1.48 × 10^−4^	9.94 × 10^−3^	107.27	121.45	24.77	167.22	3.82 × 10^−7^	0.240	–0.212
TWIP2	−0.44	2.65 × 10^−5^	6.49 × 10^−3^	207.90	592.99	66.93	2525.51	5.86 × 10^−10^	0.124	−0.043
MBIP	−0.41	1.27 × 10^−4^	9.41 × 10^−3^	58.26	71.05	13.92	109.60	6.06 × 10^−7^	0.443	−0.362

**Table 3:** EIS fitting parameters for high-Mn steels in 3.5 wt.% NaCl and 10 wt.% NaOH electrolyte solutions at 25 °C (see Figure 6), using the electrical equivalent circuit (EEC) model of Figure 8.

Specimen	*R*_s_ Ω cm^2^	*R*_film_ Ω cm^2^	*Y*_film_ µS cm^−2^ s^n1^	*n*_1_	*R*_ct_ Ω cm^2^	*Y*_dl_ µS cm^−2^ s^n2^	*n*_2_	*χ*^2^ *
3.5 wt.% NaCl								
TWIP1	28.1	607.7	0.6	0.596	2220	0.02	0.638	1.97 × 10^−3^
TWIP2	27.3	142.5	6.2	0.588	21593	0.01	0.841	6.11 × 10^−3^
MBIP	26.0	206.9	21.6	0.492	952	0.01	0.770	2.91 × 10^−3^
10 wt.% NaOH								
TWIP1	32.1	215.3	28.7	0.466	3324	0.05	0.711	4.41 × 10^−3^
TWIP2	31.2	136.6	5.4	0.607	11310	0.01	0.847	6.48 × 10^−3^
MBIP	30.1	295.1	42.4	0.536	555	0.03	0.718	8.69 × 10^−3^

* Total Error < 10%.

## Data Availability

The raw/processed data required to reproduce these findings cannot be shared at this time as the data also forms part of an ongoing study.
